# Applying the Bow Tie Method to Evaluate Emerging Risk: The Case of Carbon Capture and Water Stress

**DOI:** 10.1111/risa.70296

**Published:** 2026-06-25

**Authors:** Matt J. Weisner, Vincent P. Paglioni, Ryan P. Scott

**Affiliations:** ^1^ Department of Political Science Colorado State University Fort Collins USA; ^2^ Risk, Reliability, & Resiliency Characterization (R3C) Lab, Department of Systems Engineering Colorado State University Fort Collins Colorado USA

**Keywords:** bow tie risk assessment, carbon capture and storage, institutional fragmentation, local government, water security

## Abstract

Emerging environmental risks are often shaped not by a lack of knowledge alone, but by fragmented information across systems, disciplines, and levels of governance. This fragmentation limits the ability of local decision‐makers to identify and respond effectively to rapidly developing technologies. This paper introduces a novel bow tie risk assessment framework as a practical tool for identifying and organizing these risks. By integrating engineering, environmental, and policy perspectives, the approach captures interactions across land use, water systems, and governance structures. We apply the framework to carbon capture, utilization, and storage (CCUS) development in Colorado. Analysis shows that tracking how CCS, CCU, and CCUS are defined and applied across institutions reveals gaps in accountability, coordination, and risk identification. These inconsistencies contribute to policy drift, obscure system‐level impacts, and limit stakeholder engagement. The bow tie framework makes these gaps visible, drawing attention to risks that remain hidden within fragmented knowledge and governance systems. Findings from the Colorado case study indicate that current CCUS governance lacks consistent mechanisms to define, measure, and account for water use and impacts across institutions. The analysis highlights a critical gap in how water is conceptualized and measured, particularly where it may be permanently removed or altered through subsurface injection, storage, or disposal in ways that do not align with conventional distinctions between consumptive and non‐consumptive use. The method provides a practical tool for local and regional governments to identify risks, engage broader stakeholders, and support coordinated, interdisciplinary, and adaptive decision‐making.

## 1 Introduction

Emerging technologies present challenges in identifying reliable information early enough to reduce risks and maximize benefits (Kuzma and Priest 2010; Kates et al. 1983). While forecasting, scenario development, and quantitative risk assessment provide an established basis for early decision‐making (Cox 2012), they typically depend on data availability and expert‐driven inputs (Bates et al. 2016; Linkov et al. 2018). In areas where emerging technologies are rapidly developing with the potential to impact local communities, such as data centers, geothermal development, or battery deployment, local governments are left with limited tools to critically assess and respond to risk without first waiting on formal risk assessments from scientists or guidance from state or federal government research or review processes. Tools that translate complex information into actionable guidance are critical for effective risk governance (Linkov et al. 2018).

This paper applies the bow tie method to evaluate emerging environmental and community impacts and identify threats and consequences. This approach aligns with the qualitative variant described by de Ruijter and Guldenmund (2016), particularly the Shell HEMP model, which simplifies fault and event tree logic into communicative cause‐and‐effect relationships. The Shell HEMP model is a foundational barrier‐based risk framework used in the oil and gas sector to provide clear causal pathways for identifying hazards and controls (de Ruijter and Guldenmund 2016). Its value lies in providing a clear way to identify hazards and visualize the controls. This paper expands on the Shell Hemp model by adapting it for environmental governance and institutional risk, offering a low‐resource framework for subnational governments and non‐specialist users. Using the case of emerging carbon capture development and water stress in Colorado, we provide recommendations for practitioners on applying the bow tie method.

Carbon capture and storage (CCS) and carbon capture and utilization (CCU) are part of a broader portfolio of technologies designed to manage atmospheric CO_2_ levels. In this manuscript, CCS refers to carbon capture and storage, CCU refers to carbon capture and utilization, and CCUS is used only when referencing combined policy frameworks or documents that group these technologies together. We distinguish CCS and CCU conceptually because they involve different processes, risks, and governance pathways but use CCS/CCU as shorthand. The combined term “CCUS” can obscure these differences. Both CCS and CCU are water‐intensive processes that may disrupt hydrologic systems, especially when deployed at scale. Impacts can extend across surface water supplies, brackish aquifers, and deep saline formations (McIntosh and Ferguson 2019; Rosa et al. 2020). These pressures are particularly relevant for arid regions like Colorado, where water scarcity reflects physical supply constraints and water stress captures the impact of infrastructure, demand, and governance on that supply of, which are already shaping local government decision‐making (CEO Water Mandate 2014). Public perceptions of CCS/CCU remain limited and often negative, contributing to mistrust in large‐scale mitigation efforts (Eberenz et al. 2024; Pianta et al. 2021, p. 49). Here, the concern reflects limited public awareness and uneven understanding of CCS and CCU across governance levels and community members, rather than a lack of published research on public perceptions. In this context, risk assessment can help bridge gaps, inform stakeholders, and identify blind spots in environmental planning (Fischhoff 2007).

Colorado is a timely site for this analysis, advancing CCS/CCU policy amid water management pressures. We find that applying the bow tie framework has the potential to support integrated planning across levels of government in water‐stressed regions. We situate our analysis in Colorado's CCS/CCU water stress context, introduce the bow tie method for subnational risk management, and apply it to a literature review of CCS/CCU water threats. We conclude with recommendations for applying this approach to other emerging technologies.

### 1.1 CCU/CCS and Water Stress Uncertainty

The Intergovernmental Panel on Climate Change (IPCC) Special Report on the Impacts of Global Warming of 1.5°C (2022) identifies CCS/CCU as pivotal technologies for reducing industrial CO_2_ emissions, signaling their rise as a mainstream climate solution. CCS is designed to permanently sequester carbon dioxide in geological formations, while CCU repurposes carbon dioxide into industrial products, often resulting in delayed emissions (Bruhn et al. 2016; Thielges et al. 2022). The combined term “carbon capture, utilization, and storage” (CCUS) obscures this distinction between reuse and storage, inflating the perceived climate benefits of utilization technologies (Bruhn et al. 2016; Thielges et al. 2022). These distinctions, their implications, and potential environmental impacts are rarely discussed. Colorado, a major oil and gas producer, aims to cut emissions 100% by 2050 and positions CCS/CCU as a bridge between climate goals and economic transition (Colorado Energy Office 2024, 2025; U.S. EPA 2024). However, water impacts remain largely unexamined at the state level, including in major planning documents such as the Colorado Water Plan (Colorado Water Conservation Board [CWCB], 2023).

While Colorado positions CCUS as an innovation driver, limited attention has been given to how these technologies intersect with water, health, and local community development. At the substate level, many jurisdictions have not evaluated how CCS/CCU may affect local water resources, land use, or public health. Although some jurisdictions have begun exploring CCUS potential, a significant gap remains in assessing its systemic risks across non‐energy domains. Moreover, water stress and CCS/CCU risks are largely independent drivers, yet their interaction can create compounded vulnerabilities.

Water and energy requirements vary significantly across CCS and CCU technologies. For instance, retrofitting power plants with CCS/CCU can substantially increase water demand in water‐stressed regions such as Colorado, while certain CCS/CCU processes may also elevate air pollution and associated health risks (Jacobson 2019; Rosa et al. 2020). The pursuit of CCS/CCU thus compounds existing vulnerabilities and may increase multiple risks for Coloradans. In water‐scarce regions governed by prior appropriation, local depletion can impose significant burdens on local governments, highlighting limits in existing approaches to assessing emerging risks (Linkov et al. 2018; Rosa et al. 2020; Rosa et al. 2021). For example, Direct Air Capture (DAC) and post‐combustion CCS/CCU can increase water use by 20%–80%, while bioenergy with CCS is classified as a high water‐use technology due to irrigation and steam cycle losses (Madhu et al. 2021; Koornneef et al. 2012; Crotty et al. 2023). A consolidated comparison of environmental and water‐use impacts across CCS/CCU technologies is also summarized in Appendix A.

For governments studying CCS/CCU impacts, there is little clarity in understanding differences among technologies and their potential impacts. The lack of coherence in international CCS/CCU classification is mirrored in U.S. national policy, where inconsistencies in terminology and fragmented governance structures blur the boundaries between distinct technologies. The Department of Energy's (DOE) De‐risking CCS report (2022) regularly refers to CCS and CCUS interchangeably, particularly in discussions related to funding mechanisms and regulatory pathways. The report describes CCS as “a proven technology with the potential to stem some of the worst effects from climate change” (IV). It emphasizes economic opportunities and operational feasibility (Barrios et al. 2022). Meanwhile, major federal agencies responsible for environmental oversight, such as the U.S. Geological Survey, Bureau of Land Management, and Army Corps of Engineers, are largely absent from CCS‐related discussions.

Even Colorado's existing systems to identify risks are fragmented. The Colorado Division of Water Resources oversees water rights and scarcity, while the Colorado Energy and Carbon Management Commission (ECMC) regulates oil and gas operations, including CCS/CCU, with limited coordination between the two domains. There is virtually no integration between the Colorado Water Plan and the state's policies related to CCS/CCU development within their primary public facing documents. This disconnection is significant given the water demands and environmental risks posed by CCS/CCU technologies, particularly in a state already experiencing acute water scarcity (Colrado Energy and Carbon Management Commission [ECMC] 2024; CWCB 2023). While Colorado has made progress in water efficiency, the state has struggled to balance population growth with available freshwater. By 2050, Colorado faces a projected deficit of 230,000–740,000 acre‐feet of water, potentially outpacing all current municipal use, depending on the rate of growth and climate trends (CWCB 2023; Mullane 2024, p. 4). But growing challenges threaten to outpace existing strategies. “It is increasingly important to make sure every water project or strategy uses water as wisely as possible, making it stretch as far as it can to realize its maximum value for cities, farms, streams, and people” (CWCB 2023, p. 4). Colorado's water availability represents a wicked, ill‐structured problem that demands cross‐sector planning (MacDonnell 2015, p. 311; Rittel and Webber 1973; Simon 1955). This complexity underscores the need for integrated CCS/CCU risk assessment approaches that account for water constraints and governance fragmentation. In the absence of clear federal guidance or state policy, how can local actors, risk planners, and communicators identify risks and build resilient systems?

## 2 Governing Risk at the Policy Intersection

The bow tie model addresses two challenges: identifying and communicating risks (Pitblado and Weijand 2014). These challenges are intensified by the fact that agencies that manage water and agencies that oversee oil and gas or CCUS permitting do not share common terminology, data systems, or regulatory priorities. As a result, institutions often operate with incompatible definitions, including key terms such as “reuse” and “recycle,” and they are not integrated partners in planning or regulation. Limited technical capacity at the local level and fragmented oversight across state and federal agencies further complicate the identification and communication of emerging CCS and CCU risks. Risk assessments can serve both public agencies and private institutions by identifying critical points of vulnerability and response. The framework presented in this paper supports the development of locally integrated risk assessments. It aligns with Kline and Renn's (2021) concept of “postnormal risk governance,” which emphasizes participation, deliberation, and local legitimacy.

This is especially important when evaluating emerging technologies like CCS/CCU, where long‐term impacts remain uncertain and unevenly distributed across jurisdictions. The bow tie framework, originally developed in the process safety and engineering fields in the late 20th century, merges fault tree and event tree analysis into a visual structure for identifying hazards, controls, and consequences (Pitblado and Weijand 2014; de Ruijter and Guldenmund 2016). It has since been adopted in high‐risk domains such as aviation, oil and gas, and chemical safety to assess both technical and institutional vulnerabilities (Angel and Jayaparvathy 2023).

Building on this broader literature, this paper applies the bow tie method to environmental governance. In this context, the framework helps anticipate coordination failures and supports a shift from reactive to anticipatory planning by highlighting not only physical risks but also governance gaps that require structured response.

The bow tie framework has been widely used as a heuristic for understanding the independent threats facing engineered and sociotechnical systems, the preventive and mitigative barriers in the system, and the consequences resulting from a realized threat (de Ruijter and Guldenmund 2016). In a Web of Science search, using search terms (“bow tie” AND “risk”) in all document fields yielded 252 publications incorporating the bow tie risk assessment framework since 2021, distributed across broad fields according to Table 1. While variations on the bow tie framework exist, the left side (converging section) generally expresses the threats (causes) and preventive barriers in place to prevent the occurrence of the central event, while the right side (diverging section) represents the possible consequences and available mitigative barriers. In engineering applications, the bow tie method is often combined with more quantitative approaches, such as fault trees (Angel and Jayaparvathy 2023), event trees (Barros et al. 2021), and/or Bayesian networks (Tong et al. 2025).

**TABLE 1:** Distribution of publications using bow tie risk assessment by field (2021–2025).

Field	Articles
Automation and control, computer science	20
Business and economics	9
Energy and physical sciences	40
Construction and building technologies	8
Engineering	136
Environmental science and health	39

This table summarizes findings from a Web of Science search of 252 publications using the terms “bow tie” and “risk” across all document fields. Publications may appear in more than one category because many span multiple disciplinary domains or applications.

However, the explainability of the bow tie framework and its value as a heuristic for understanding system risk points to its broader applications. In this study, the bow tie framework is constructed from systematically coded documentary evidence drawn from peer‐reviewed literature, government reports, regulatory filings, and organizational documents, rather than direct expert elicitation approaches commonly used in risk governance (Linkov et al. 2018, p. 173).

The bow tie method supports collaborative and interdisciplinary risk governance, particularly as a tool for communication and stakeholder engagement (Renn 2008; Linkov et al. 2018). For example, when a local government applies the bow tie framework to an emerging CCS or CCU proposal, it may identify foundational gaps such as uncertainties in water demand, incomplete reporting systems, or inconsistencies between water‐sector and oil and gas sector definitions identified in this study. As the assessment is applied to specific sites, additional information needs may emerge, and some risks may become more or less relevant depending on local conditions.

This research leverages the inherent explainability of the bow tie structure and the utility derived from constructing the bow tie to facilitate higher level risk decisions with different stakeholders. That is, while local and state‐level officials are not the typical audience for risk assessments, they are often key stakeholders in risk‐informed decisions. The bow tie framework developed herein ensures that decision‐makers have a sound, approachable, and useful tool for conceptualizing and visualizing system risk.

Local communities possess the most relevant knowledge for identifying site‐specific vulnerabilities (Ostrom 1990; Nsoh 2022); thus, the method is intentionally low‐resource and designed for non‐specialists such as municipal staff, relying on publicly available documents and a simple spreadsheet. As de Ruijter and Guldenmund (2016) caution, complex frameworks must be adapted to their implementation context. As a qualitative variant, the bow tie method can help non‐specialists identify environmental risks and flag areas where expert input is warranted. CCUS then serves as an ideal case for demonstrating the benefits of the bow tie method given ambiguity and uncertainty in not only what CCUS is but also what its likely impacts on stressed local resources may be. CCS and CCU present an especially suitable case for applying the bow tie framework because these technologies interact directly with stressed and variable water resources, requiring local governments to interpret potential impacts with limited information and uneven planning capacity. CCS and CCU also operate at the intersection of energy, environmental protection, and water allocation, which highlights the operational fragmentation that this framework is designed to clarify. The case is additionally timely because Colorado's permitting and oversight structures for CCS and CCU are still emerging, making early identification of risks and uncertainties essential for effective governance against a backdrop of persistent water‐quantity and developing water‐quality concerns.

## 3 Methods

This study applies a bow tie risk assessment to evaluate the effects of CCS/CCU on local water systems in Colorado. The single‐case design examines how interactions between emerging technologies and water resource management generate potential risks, drawing on peer‐reviewed literature, government reports, regulatory filings, and other primary sources. Before the literature review, we examined how CCS, CCU, and CCUS are defined and framed in international policy discussions, forming the basis of the dominant policy narrative (Langhelle et al. 2024). This step clarifies what is being assessed and why certain risks may be overlooked or diminished.

Our top event, the principal risk event addressed in this analysis, is water scarcity from CCS/CCU implementation. We conducted a literature review of 16 documents published between 2012 and 2025, with a focus on sources from 2020 onward to reflect current scientific and policy developments. Sources included peer‐reviewed articles, government reports, regulatory filings, and independent news sources, identified through systematic keyword searches: (CCUS or CCS or CCU) and (water or scarcity) and (lifecycle or review). Review and lifecycle articles were included for their synthesis of technical and policy trends.

The publicly accessible sources (Appendix A; Weisner 2025) provided the empirical foundation for identifying environmental threats and policy gaps across three analytical goals: (1) assessing CCS/CCU water risks; (2) examining how policy language affects risk framing and identification; and (3) deriving insights from related technologies, including oil and gas extraction, ethanol production, and deep well injection. Attention was given to threats to current and future drinking water supplies related to consumption and contamination.

Documents were evaluated for their relevance to risk pathways, mitigation strategies, and institutional barriers and were coded for (1) risks, (2) barriers, and (3) policy gaps (Appendix A; Weisner 2025). Coding was guided by predefined categories derived from the bow tie framework and applied as a structured, framework‐guided interpretive process to organize documentary evidence. Coded entries include direct quotations from source materials to ensure traceability and allow verification of how interpretations were derived. Where necessary, entries retain extended quotations to preserve context and provide a transparent audit trail linking source material to analytical classifications. The goal of coding is not data reduction but the structured translation of documentary evidence into components of the bow tie framework. To clarify how the documentary evidence was translated into the bow tie structure, a five‑step analytic workflow:

1. Define the hazard and top event: CCS/CCU water impacts were identified as the hazard, with “water scarcity” as the top event.
2. Gather relevant documents: Systematic keyword searches identified peer‐reviewed literature, government reports, regulatory filings, and institutional documents related to CCS, CCU, and water governance.
3. Code documents: Relevant segments of documentary evidence were systematically coded using a structured template aligned with bow tie components to identify risks, barriers, consequences, and governance gaps.
4. Map coded content to bow tie components: Coded segments of documentary evidence were mapped to threats, preventive barriers, mitigative barriers, and consequences.
5. Synthesize the bow tie model: The resulting components were integrated into a bow tie diagram representing causal pathways and governance responsibilities.

To structure this analysis, the bow tie method was applied as a qualitative risk assessment framework, following Pitblado and Weijand (2014). This approach organizes risk around a top event, mapping causes, barriers, and consequences in a structured way. The framework defines the following elements:

- Hazard: CCS/CCU activities with the potential to disrupt water security.
- Top Event: Water scarcity resulting from CCS/CCU implementation.
- Causes: Initiating threats such as water quality degradation, freshwater over‐extraction, and regulatory uncertainty.
- Barriers to Causes (Prevention): Policies, monitoring, and mitigation technologies that prevent hazards from escalating.
- Barriers to Consequences (Mitigation): Risk response mechanisms that reduce the severity of CCS/CCU water impacts.
- Consequences: If barriers fail, outcomes include water contamination, depletion, and long‐term regulatory challenges.

In the bow tie framework, governance mechanisms appear on both sides of the top event: as preventive controls (e.g., permitting and coordination) and as mitigation strategies (e.g., enforcement and contingency planning). Highly cited sources were prioritized to ground the assessment, while emerging risks and gaps in knowledge were also considered. The model is used as a design structure to organize current knowledge and can be updated as new empirical data becomes available. All 16 documents are publicly accessible (Appendix A; Weisner 2025).

## 4 Results

The policy narrative review shows that CCUS is not a neutral or technical term but a policy label that combines different technologies with vastly different outcomes. This framing has leveraged an existing gap between climate mitigation goals and economic drivers, as well as between energy and water governance, reinforcing institutional silos and complicating integrated risk assessments. The shift from CCS to CCUS began around 2012 and became more prominent in international climate discourse by 2014, particularly in the IPCC's Fifth Assessment Report. Since then, CCUS has been widely adopted in policy and industry, often without clear distinctions between storage and utilization, contributing to persistent governance challenges (Langhelle et al. 2024; Bruhn et al. 2016; Thielges et al. 2022).

Grounded in the compiled sources (Weisner 2025) and structured using the bow tie framework (Pitblado and Weijand 2014), the analysis maps hazards, threats, and mitigation strategies to evaluate cascading risks. The results highlight key concerns related to CCS/CCU, including water availability, permitting gaps, and weak coordination among agencies. Rather than calculating probabilities, this approach focuses on how risks interact and where governments at various scales can intervene along risk pathways.

### 4.1 Hazard: Ccs/Ccu

In the bow tie model, a hazard is defined as an activity with the potential to lead to a top event. For CCS/CCU, the hazard lies in their substantial water requirements and the contamination potential during operations, transport, and storage. Without comprehensive performance standards or oversight, these hazards can result in long‐term degradation of water resources. CCS/CCU, therefore, require a well‐defined barrier management system.

**FIGURE 1:**
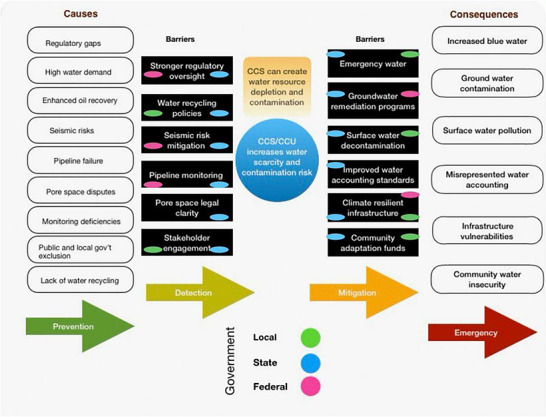
Barrier bow tie diagram illustrating water resource management challenges. This bow tie risk assessment highlights CCS/CCU impacts on water resources. It visualizes key threats, prevention barriers, and mitigation strategies across governance levels. Without intervention, groundwater contamination and water insecurity risks may escalate. Strengthened oversight, stakeholder engagement, and adaptive planning are critical to managing these challenges.

Figure 1 illustrates the threats, consequences, and responses associated with CCS/CCU deployment in Colorado. The diagram maps key failure pathways related to high water demand, regulatory uncertainty, and induced seismicity. These conditions increase the likelihood of water scarcity and contamination. Without strong preventive controls such as water use regulation and recycling standards, the system is vulnerable to escalating impacts. Intervention must shift across governance levels depending on where the failure occurs.

The bow tie framework used in this study (see Figure 1) organizes risk into four phases: prevention, detection, mitigation, and emergency response. The diagram maps threats, barriers, and consequences across governance levels, with responsibilities distributed among local, state, and federal actors. As risks move from causes to consequences, local governments increasingly carry the burden, especially when early barriers fail.

On the left, the diagram outlines root causes such as regulatory gaps, high water demand, and limited monitoring. In the center, barriers are shown across governance levels, including tools like stronger oversight, water recycling policies, and stakeholder engagement. On the right, consequences reflect escalating impacts when risks are not effectively managed, including groundwater contamination, infrastructure failure, and community water insecurity. In water‐scarce states like Colorado, addressing these gaps are critical to managing the long‐term risks of CCS/CCU.

Prevention aims to avoid the top event, defined here as accelerated water stress. Local governments may pursue land use controls, such as zoning restrictions, though these are often superseded by state‐level siting decisions. State prevention could include water use performance standards for CCS/CCU, but such measures remain largely absent. At the federal level, prevention may occur through the integration of water risks in National Environmental Policy Act (NEPA) reviews and Environmental Protection Agency (EPA) Class VI well permitting. Colorado is currently pursuing primacy over the federal Class VI program, which would transfer this responsibility to the state (ECMC, 2024). While primacy may enhance regulatory control, it can also expand governance responsibilities and shift risk management burdens across state and local institutions, as responsibilities are redistributed across agencies with overlapping roles, potentially weakening preventive consistency.

Detection identifies early signs of system stress. At the local level, this may involve community‐based monitoring, particularly in agricultural districts. At the state level, real‐time water use reporting is an essential but underdeveloped detection mechanism. At the federal level, the EPA and the Pipeline and Hazardous Materials Safety Administration (PHMSA) share detection responsibilities, though PHMSA has not yet updated pipeline rules despite recent CO_2_ pipeline failures, as discussed below.

Mitigation seeks to reduce the severity of outcomes after the top event has occurred (i.e., after CCS/CCU increases water scarcity). Local governments may adopt contingency plans and establish remediation funds. State mitigation includes strengthening plugging and abandonment rules regarding wellheads and establishing bonding requirements for CO_2_ infrastructure. At the federal level, historical tools include liability frameworks and remediation grants, though their adequacy remains in question.

Emergency responses are activated when consequences are near or fully realized. Local responders manage immediate threats to aquifers or pipeline failures. States may deploy mobile testing units and emergency water reserves. Federal involvement typically follows, led by FEMA, PHMSA, or the EPA as part of coordinated disaster response or as an after‐action review.

Effective risk governance requires multilevel coordination across all phases of the bow tie framework. Each government level plays a unique role, but alignment across jurisdictions is essential for managing cumulative risks. This is critical in water‐stressed regions like Colorado, where CCS/CCU infrastructure projects have far‐reaching and uneven impacts.

### 4.2 Top Event—Accelerating the Water Stress

In the bow tie framework, the top event represents the point at which prevention measures have failed, and the system enters a stage of managing escalating consequences. In this case, the top event occurs when CCS/CCU implementation exacerbate water scarcity due to their high operational water demands. Uncontrolled water withdrawals for CCS/CCU create cascading risks, especially in arid regions where water is already contested among industrial, municipal, and agricultural users. The effectiveness of prevention barriers depends on whether water use thresholds, monitoring frameworks, and response strategies are established and maintained before critical water stress or scarcity occurs.

### 4.3 Initiating Threats

Systemic threats such as regulatory uncertainty, geophysical instability, and the absence of long‐term planning increase the likelihood that CCS/CCU will intensify pressure on water supplies. Additional threats that could result in the top event include weak oversight, unresolved liability, and limited monitoring capacity. CO_2_ separation and injection also often require large volumes of water, directly reducing availability for other uses (Bruhn et al. 2016; IPCC 2022; Thielges et al. 2022). Many jurisdictions lack enforceable water‐use thresholds or obligations for CCS operators. Geological constraints limit alternative site management strategies, while industry‐driven expansion increases stress on local water systems. If unaddressed, these threats can intensify water competition, increase contamination risks, and reduce overall system resilience. Dry‐year and low‐flow conditions identified in the Colorado Water Plan function as hydrologic escalation factors that weaken preventive and detection barriers and increase the likelihood that CCS/CCU water impacts exacerbate water stress.

### 4.4 Barriers to Prevent the Top Event

Effective prevention barriers are essential to managing CCS‐related water risks. Lessons from oil and gas operations show that regulatory enforcement, advanced monitoring, and technical safeguards can help prevent systemic failures. However, as extraction efforts have moved to target deeper geological formations, risks have also evolved, demanding increasingly sophisticated oversight. Similar protections and adaptive mechanisms are largely absent for CCS/CCU policy design regarding water resources. Current groundwater monitoring networks are insufficient, providing little real‐time data on withdrawals or contamination. The absence of validated risk models hinders forecasting of CO_2_ migration and long‐term impacts on water availability. Strengthening well integrity rules is critical, but without clear performance indicators for sustainable water use, enforcement remains weak. Without these safeguards, CCS/CCU may proceed unchecked, escalating stress on freshwater systems and exposing communities to avoidable harm.

### 4.5 Barriers to Mitigate Consequences

In bow tie risk management, mitigation barriers reduce the severity of outcomes once the top event has occurred. While plugging abandoned wells can reduce fluid migration, inconsistent implementation and prohibitive costs undermine compliance. Injection‐extraction balancing could help manage reservoir pressure and reduce leakage risk but remains underused (Kazlou et al. 2024; Koornneef et al. 2012). Enhanced groundwater monitoring can support early detection of contamination, though underfunded systems remain a persistent barrier. Insights from industrial safety practices emphasize the need for geophysical surveys, leak detection, and liability frameworks, yet these are weakly developed for CCS/CCU, leaving affected communities and ecosystems exposed.

### 4.6 Consequences

Consequences represent outcomes if both preventive and mitigation barriers fail. In the context of CCS/CCU, these include groundwater contamination, long‐term water depletion, and governance breakdowns. Unchecked withdrawals can threaten agricultural production, municipal supply systems, and ecosystem stability in water‐scarce regions. Regulatory gaps exacerbate these risks, as few jurisdictions mandate water tracking or transparent reporting for CCS/CCU operations. Without sustained oversight, adverse impacts may go undetected for years, leading to litigation, public distrust, and unresolved environmental liabilities. These outcomes underscore the need for stronger preventive and mitigative controls before impacts become irreversible, ensuring that CCS/CCU does not undermine critical water resources.

## 5 Key Considerations for Local Governments

CCS and CCU present governance challenges that require coordinated action across local, state, and federal agencies to ensure that the potential benefits are realized while risks are effectively managed. These technologies are already being pursued in Colorado and elsewhere, which means local governments are encountering risks and uncertainties in real time rather than in a hypothetical future. This section therefore highlights the institutional considerations that shape how CCS and CCU can be deployed in ways that protect water resources, support community priorities, and align with long‐term planning goals. First, local jurisdictions must balance economic incentives against environmental protection. Tax credits or subsidies often drive CCUS approval, but reinvestment is needed to reduce risks. Financial gain can become a lever for initiative‐taking governance. Without reinvestment, CCS/CCU projects risk amplifying water scarcity and ecological vulnerability. This finding is supported by the CCS/CCU Governance & Water Bow tie Evidence Table (Weisner 2025), a coded dataset linking sources to risk elements; for example, Ning et al. (2025) was coded under ‘causes’ for showing how 45Q tax credits accelerate CCUS without water safeguards.

Second, regulatory oversight must be strengthened at all levels. Local governments require better integration of permitting processes, zoning powers, and environmental protections to ensure CCS/CCU projects align with long‑term water and land‑use planning. This includes anticipating potential land‑use conflicts, especially in agricultural or peri‑urban areas, and developing policies that support early coordination with state and federal agencies. The evidence table places ECMC's Safety Study under barriers not because it offers protections but because it highlights the absence of them, including missing water‑use regulations and measures to prevent net water loss. These gaps may compound land‑use conflicts when CCUS projects compete with agriculture for limited water.

### 5.1 Colorado Water Governance and Measurement Fragmentation

Similarly, the Colorado Water Plan is coded under hazard because it recognizes water scarcity as a systemic risk; however, neither the CWCB (2023) nor ECMC (2024) safety study explicitly evaluates net water loss or water permanently removed from future beneficial use through CCUS‐related pathways. Third, stakeholder engagement and transparency are essential. Public perception can significantly influence project approval (Pianta et al. 2021; Eberenz et al. 2024). Inconsistent information, inaccessible technical documents, and limited opportunities for public engagement may erode trust and delay critical decisions. Although communities are often given opportunities to engage with CCS and CCU projects, the information provided may be incomplete, inconsistent, or difficult to access, limiting their ability to fully understand the associated risks and benefits, particularly in water‐stressed regions. Evidence from the literature, including Hall (2024) and Kaifang et al. (2024), indicates that poor communication and lack of transparency increase opposition and delay CCUS projects, reinforcing the need for accessible information and inclusive public forums.

Fourth, water risks from CCS and CCU remain partially addressed within existing regulatory frameworks. Water withdrawals for CO_2_ capture and injection, the potential for aquifer contamination, and legacy wellbore leakage present serious challenges for local governments tasked with local response. While regulatory frameworks include provisions for corrective actions and remediation, scientific literature indicates that complete remediation of groundwater systems is often unlikely once contamination occurs due to the complexity and uncertainty of subsurface processes, even with substantial funding (U.S.EPA 2013; Madhu et al. 2021). This highlights the underdevelopment of mitigation planning in CCS/CCU governance. Without robust contingency strategies, consequence management relies on optimistic assumptions that may not reflect long‐term risks.

A key gap in Colorado's water governance, as described in the Colorado Water Plan (CWCB 2023), is the lack of clear definitions and tracking for water that is effectively and intentionally removed from future beneficial use through subsurface disposal or sequestration. The plan defines consumptive use as “any use of water that permanently removes water from the natural stream system and water that has been evaporated, transpired, or incorporated into products, plant tissue, or animal tissue and is not available for immediate reuse” (p. 241). While this definition traditionally applies to agricultural and municipal uses, industrial activities such as oil and gas wastewater disposal, sewage injection, and CCS/CCU introduce a distinct challenge: water may be placed into deep subsurface formations specifically to isolate it from recovery, circulation, or reuse, yet these losses are not consistently accounted for or discussed as reductions in available freshwater supply.

ECMC applies “water reuse” and “recycle” as operator‐reported waste management concepts, focusing on treatment and operational handling of produced water. In contrast, the Colorado Water Plan frames these concepts in terms of consumptive use and depletions by emphasizing whether water is permanently removed from the stream system or remains available for reuse to downstream uses (CWCB 2023, pp. 51–55). Much of this produced water is permanently removed, yet current regulatory language does not fully capture this distinction.

This distinction is reinforced by HB23‐1242, which requires operators to report volumes of fresh, recycled, reused, and disposed water and indicate when water is ‘not returned to the hydrologic cycle’ (ECMC, 2024). ECMC rules reinforce this through Fresh Water Plans (Rule 304.c.(18)) and reuse/recycling plans (Rule 905.a(3)). Regulation 84 addresses reclaimed water for non‐potable uses but does not account for permanent removal, risking underestimation of losses critical to long‐term supply (CWCB 2023). Closing these definitional gaps is essential for accurate water budgeting and aligning energy‐sector practices with Colorado's conservation goals. Without integrating hydrologic concepts into operational rules, this misalignment creates a systemic blind spot in Colorado's water accounting, where the reporting of recycled or reused water may overstate conservation benefits when the share of total water use actually met through reuse remains unknown. This problem is compounded by the fact that produced water's high salinity and contamination make reuse more difficult and costly and may further obscure the true scale of permanent freshwater losses.

## 6 Discussion

The bow tie framework helps local governments identify emerging risks and coordination gaps. By visually organizing hazards, threats, and mitigation strategies, it clarifies where uncertainties persist and where institutional barriers may be misaligned. The adaptive dimension of the bow tie model. As new evidence emerges, local governments can update assessments and planning tools, fostering transparency and accountability. This aligns with broader approaches to participatory and adaptive risk governance (Klinke & Renn 2021).

More broadly, the bow tie framework functions as a practical workflow tool for local governments that often lack the technical capacity or formal authority to direct CCS and CCU permitting. It helps trace how risks unfold and where responsibility shifts across governance levels, revealing where coordination gaps are most likely to occur. This clarity helps local officials anticipate needed oversight, coordination, and contingency planning.

The visual layout also supports non‐specialists by highlighting early indicators and prompting questions about missing information, such as water‐use data, monitoring requirements, or site‐specific safeguards. This helps local planners to anticipate necessary contingency measures. Because the bow tie can be updated as new policies emerge, it supports a more adaptive approach to managing CCS and CCU risks.

A persistent challenge in CCS/CCU governance is the global–local divide. Climate mitigation is coordinated at the global level, while water management remains a local responsibility. This structural gap complicates policy alignment, but the bow tie framework helps bridge it by providing a shared visual language for stakeholders across governance scales. Public trust remains fragile in CCS/CCU governance, making transparent communication critical (Slovic 1993).

An emerging example of regulatory misalignment is the use of CO_2_‐based hydraulic fracturing in oil and gas operations, often marketed as “waterless.” This framing is misleading because it obscures subsurface hazards and aquifer contamination risks (Silva‐Escalante et al. 2025; Koornneef et al. 2012; McIntosh and Ferguson 2019). In the United States, these operations are typically permitted under Class II wells designed for hydrocarbon production, even though their risks resemble those associated with Class VI wells for CO_2_ sequestration (Kazlou et al. 2024). Both Class II and Class VI wells fall under the Safe Drinking Water Act (SDWA), meaning CO_2_ fracturing is not truly “waterless.” Nonetheless, regulatory practice and industry narratives continue to reinforce this misconception.

The expanding CO_2_ pipeline network raises public health and emergency response concerns, as shown by the Satartia, Mississippi CO_2_ release (PHMSA 2022). These inconsistencies reflect a broader regulatory lag, where climate‐linked technologies are scaled without integrated oversight. Misleading labels and fragmented governance obscure structural vulnerabilities in both water management and carbon regulation, revealing how mitigation narratives can unintentionally increase local risks. As CCS and CCU deployment accelerates, improving coordination between local, state, and federal actors will be critical. Without such alignment, the burden of oversight and emergency response will continue shifting to local governments, which often lack the technical capacity, funding, or authority to manage these complex risks alone. These patterns highlight persistent regulatory blind spots and underscore the need to anticipate emerging risks as new technologies and policy narratives evolve.

### 6.1 Uncertainties and Policy Drift

First, there is significant epistemic uncertainty around the implementation of CO_2_ sequestration projects that will affect the typology and manifestations of risks, available and effective barriers, and the affected domains. This epistemic uncertainty stems from a lack of completed CCS/CCU projects, defined as a site where injection has ended, long‐term containment has been verified, and active monitoring is no longer required, from which to draw conclusions about implementation approaches and long‐term safety. Recent geochemical monitoring research shows that natural variability in groundwater chemistry and limited baseline datasets can make it difficult to distinguish CO_2_ impacts from background conditions. This reinforces epistemic uncertainty and may hinder timely oversight and intervention, as early‐stage impacts may go undetected while natural variability may also trigger unnecessary concern (Littlefield et al. 2026).

Policymakers and regulators are expected to make major decisions without real‐world data proving that stored CO_2_ will remain safely sequestered. This epistemic uncertainty is expected to resolve as CCS/CCU systems mature and more data becomes available. Additionally, expanding the ability of decision‐makers to conduct risk assessments for emerging technologies enables their broader deployment, and hastens the resolution of epistemic uncertainties.

Another large source of uncertainty in CCS/CCU risk assessment is driven by evolutionary and uncertain federal carbon policies. Current trends increasingly favor CCU projects that offer immediate market returns, particularly in sectors like enhanced oil recovery and industrial feedstocks. As Langhelle et al. (2024) note, this shift reflects a strategic emphasis on commercialization, with utilization framed as a way to create a business case for carbon capture. However, this framing may obscure long‐term mitigation priorities and limit support for emerging technologies like DAC.

Although DAC is not operationally considered a form of CCU, it is often included in CCS policy frameworks when paired with geological storage (DACCS), though its classification remains debated (Langhelle et al. 2024). This ambiguity is further complicated by shared infrastructure, such as pipelines, which can obscure distinctions between utilization and storage pathways. This creates a policy drift risk, where short‐term economic incentives outpace long‐term environmental safeguards. As federal oversight continues to evolve, state and local governments will face greater responsibility for protecting water resources and managing CCS/CCU‐related risks. The transient nature of federal policies could be incorporated into the bow tie framework as a threat to “Policy” barriers, as preventive or mitigative policies may evolve to be weaker, thus potentially exposing greater risks. To a certain extent, this is an aleatory uncertainty, as federal policies are always subject to change, but it can be partially addressed by using the bow tie method to guide structured scenario analysis under different policy assumptions, drawing on robust decision‐making approaches for deep uncertainty (Lempert 2019).

Finally, water consumption accounting in CCS/CCU project planning remains poorly defined, masking risks to water use and presenting a fundamental uncertainty for the bow tie approach as it relates to safeguarding water resources. Without stronger standards, local water managers face gaps in tracking withdrawals linked to CCS/CCU deployment. Thus, a critical roadblock in the wider adoption of CCS/CCU risk assessment methods, including the bow tie approach, is the creation and adoption of standardized approaches to water consumption accounting.

These uncertainties fall largely outside the scope of the bow tie framework presented here because they are not discrete, observable threats with clearly defined causal pathways; instead, they reflect systemic gaps in data (implementation/before science), policy alignment, and institutional capacity (economics over climate and water). However, these uncertainties can be partially resolved through the application of the bow tie model itself and/or the continued collection of CCS/CCU data.

The framework created in this study is intended to serve as a science‐based foundation that can be built upon over time and continually critiqued. It is designed not only to map known risks but also to function as a visual communication tool and shared language that brings emerging uncertainties into the conversation. Because risk is perspective‐based, each of the unresolved uncertainties identified, such as policy drift, infrastructure expansion, and gaps in water accounting, can be reframed as potential threats within the bow tie structure and therefore must be actively engaged with. As new data and experience become available, these uncertainties can be modeled, measured, and addressed through updated risk pathways. This adaptability is the very thing that gives the bow tie method its real power. It does not simply organize what is known; it reveals what is missing in a particular context. In doing so, it invites ongoing inquiry and supports the development of more responsive governance systems, including the potential for direct communication channels between local and international governance bodies.

## 7 Conclusion

The bow tie framework applied to CCUS deployment in Colorado's water systems reveals that institutional fragmentation is the critical systemic risk that heightens the potential for water scarcity. As an instrument, the bow tie method makes potential risks and barriers visually assessable while providing an underlying document‐based framework for further research. It can also reveal when key decisions are being made beyond the control of local jurisdictions, prompting communities to advocate for stronger state‐level rulemaking or to open new channels of communication across governance levels to expand the conversation.

This framework serves as an operational tool to give local governments greater agency in CCS/CCU risk decisions.

To clarify how this framework operates in practice, the following summarizes its key features and how they assist local decision makers before outlining the core components. The framework developed in this study builds on the established bow tie method and incorporates additional components, including a documentary evidence coding workflow, governance level mapping, and clarification of CCS, CCU, and CCUS definitions drawn from international policy documentation. These additions make the framework suitable for subnational governance. Local governments can use it to identify missing information, understand where responsibility shifts between governments and agencies, and anticipate how risk may escalate if preventive or mitigative measures are unclear. A central example highlighted in the study is the tension between global climate goals, economic incentives that accelerate CCU development, and the local water and governance risks that communities must manage. By making these pressures visible, the framework helps planners determine when additional information, such as clearer water use projections or coordination requirements, is needed before state permitting proceeds. These applications show how the framework developed here extends the bow tie method into a practical tool that supports anticipatory, transparent, collaborative, and proactive CCS and CCU decision‐making, and the framework can also be applied to other emerging technologies that create local resources and/or governance pressures, such as data centers or geothermal development.

Visually organizing and clarifying information and language around threats, barriers, and consequences to build a cohesive understanding of the risk space across governance levels. This approach is usable by non‐specialists and municipal staff and can be linked to public documents and spreadsheets to maximize data usability.

Mapping governance responsibilities to specific barriers, color‐coded by level of government and position to distinguish prevention from mitigation, provides a clear delineation of responsibilities and facilitates effective cross‐agency cooperation and decision‐making. Making the model visible to and usable by relevant stakeholders helps minimize siloing and incentivizes coordination across agencies and levels of government.

Structuring risks and management strategies into four phases (prevention, detection, mitigation, and emergency response) provides multiple distinct opportunities to address risks as they arise. Further, this clearly identifies for decision‐makers when various risk management options are warranted and effective.

Incorporating localized data ensures that permitting, operations, and emergency response decisions are appropriate for the operating context. Because the model is adaptable to changing data sources and streams, risk‐informed decision‐making is adaptable to changing environments and locality needs.

Engaging stakeholders and local decision‐makers more meaningfully in risk decisions strengthens regulatory oversight and monitoring across governance levels. This helps to bridge the global‐federal‐local policy divides, identify institutional fragmentation, and facilitate greater local and regional agency and oversight. The inherent clarity of the bow tie approach further provides greater communication transparency to strengthen trust in decisions among stakeholders and the public.

Supporting anticipatory planning for water‐stressed regions opens additional opportunities for prevention and mitigation beyond permit‐by‐permit decision‐making. Because the bow tie approach can be updated as new data and evidence emerge, it remains adaptable to changing environmental, governance structure, and technological developments.

In this way, the bow tie method not only supports more resilient and inclusive environmental governance but also democratizes risk assessment by making it accessible to those most affected by emerging technological risks. While the bow tie method offers a valuable framework for navigating uncertainty and risks, it is important to emphasize that elements of both CCS and CCU may be needed to help meet climate goals. However, where and how these technologies are implemented must be critically examined at each level of governance. As CCUS scales, local governments on the front lines of water management and land use need the informational tools and institutional support necessary to weigh the tradeoffs, anticipate unintended consequences, and ensure the deployment aligns with community priorities and environmental resilience.

## Funding

The authors have nothing to report.

## Conflicts of Interest

The authors declare no conflicts of interest.

## Supporting information




**Supplementary Table 1**: Technology risk characteristics by sector, water demand, energy demand, and rationale
**Supplementary Table 2**: Water impact increase for selected carbon removal technologies (Rosa et al., 2020)

## Data Availability

The full evidence dataset supporting this analysis is available as Supporting Information and openly available in Zenodo at https://doi.org/10.5281/zenodo.16769145.
