# Supplementary material for: Applying the Bow Tie Method to Evaluate Emerging Risk: The Case of Carbon Capture and Water Stress
Source: Risk Anal. 2026 Jun 25;46(7):e70296. doi: 10.1111/risa.70296 (PMC13297025; doi:10.1111/risa.70296)
Supplement: Supplementary file 1 — Supplementary Table 1: Technology risk characteristics by sector, water demand, energy demand, and rationale Supplementary Table 2: Water impact increase for selected carbon removal technologies (Rosa et al., 2020) [file RISA-46-0-s001.docx]

**Appendix A: Supplementary Tables and Notes**

**Table 1:**

**Technology risk characteristics by sector, water demand, energy demand, and rationale**

| **Technology type** | **Sector** | **Water demand** | **Energy demand** | **Rationale summary** | **Source(s)** |
| --- | --- | --- | --- | --- | --- |
| **DAC (solid sorbent)** | **Cross-sector** | **Moderate (20–30%)** | **High** | **Temp swing; water for cooling/regeneration** | **Madhu et al. (2024); Rosa et al. (2023)** |
| **DAC (liquid solvent)** | **Cross-sector** | **High (up to 40%)** | **High** | **Solvent cycling; water-intensive regeneration** | **Madhu et al. (2024)** |
| **DACCS (TSA)** | **Cross-sector** | **High (+42%)** | **High** | **Temp swing adsorption; elevated water use** | **Rosa et al. (2023)** |
| **DACCS (PSA)** | **Cross-sector** | **Moderate (+32%)** | **High** | **Pressure swing adsorption; elevated water use** | **Rosa et al. (2023)** |
| **BECCS** | **Forestry, bioenergy** | **High (575 m³/t CO₂)** | **Moderate** | **Biomass combustion; blue water for capture** | **Rosa et al. (2023)** |
| **CCS (power sector)** | **Electricity** | **High (32–93%)** | **Moderate** | **Post-combustion; prolongs fossil generation** | **Koornneef et al. (2012)** |
| **CCS (natural gas)** | **Electricity, industrial** | **Moderate (qual.)** | **Moderate** | **Reuse for EOR; uncertain storage feasibility** | **Koornneef et al. (2012); Kazlou et al. (2024)** |
| **CO₂-EOR** | **Oil and gas** | **Variable** | **High** | **CO₂ used to extract oil; high recovery potential but limited deployment success due to site-specific feasibility and infrastructure gaps** | **Kazlou et al. (2024)** |
| **Pipeline transport** | **Cross-sector** | **Low** | **Low** | **Depends on source/destination; lacks release models** | **Koornneef et al. (2012)** |

Note.

1. All technologies listed are designated as potential threats in the BowTie framework due to water intensity. Also energy demand/type has a potential to compound water stress if deployed at scale. For pipeline transport, risk designation reflects uncertainty in CO₂ release modeling and route-specific exposure.

2. Most carbon capture technologies require downstream routing to either permanent storage (CCS) or industrial reuse (CCU). While some configurations, such as DAC or post-combustion capture, can theoretically feed either pathway, their mitigation value depends on geological sequestration availability. BECCS, by contrast, is a carbon removal pathway that relies exclusively on permanent storage. It is not deployable as a CCU technology, as reuse of captured CO₂ would negate its mitigation value. CO₂-EOR is a reuse application, not a storage solution. Although some injected CO₂ may remain trapped underground, this partial storage is incidental, site-dependent, and not guaranteed. CO₂ loss is inherent to the EOR process due to production cycling, reservoir inefficiencies, and operational priorities focused on oil recovery. While projects like the Bakken Field Lab and the Alberta Carbon Trunk Line aim to pair EOR with long-term CO₂ storage, publicly available data on verified retention remains limited, underscoring the gap between theoretical potential and demonstrated mitigation value.

3. Blue water refers to surface and groundwater withdrawn from rivers, lakes, reservoirs, and aquifers for human use. This includes water used for cooling, biomass irrigation, and industrial processes in CCS facilities. It does not include rainwater stored in soil or recycled water.

4. “High (575 m³/t CO₂)” indicates that capturing one tonne of CO₂ via BECCS consumes about 575,000 liters of bluewater, including withdrawals for cultivation, processing, and capture.

5. Water and energy demand for pipeline transport depend heavily on route length, elevation changes, and compressor station design. Similarly, energy demand for CCS technologies varies depending on the type of energy used and whether indirect water use (e.g., cooling systems, upstream electricity generation) is included in the accounting.

6. TSA (temperature swing adsorption) uses heat to release CO₂ from solid sorbents and typically requires high energy input and cooling water. PSA (pressure swing adsorption) relies on pressure changes to separate CO₂ and is less water-intensive but still energy-intensive. BECCS (bioenergy with carbon capture and storage) captures CO₂ from biomass combustion or processing and requires substantial blue water for cultivation and capture.

7.  Some information in Table 1 was developed from the author’s open-access dataset archived in Zenodo (XXXXXX, 2025), which includes risk framework classifications, implementation details, and citation mappings derived from published sources. All cited publications are listed in the manuscript’s reference section.

**Table 2**

**Water impact increase for selected carbon removal technologies (Rosa et al., 2020)**

| **Capture Configuration** | **Combustion phase** | **Water impact increase** |
| --- | --- | --- |
| Amine absorption | Post-combustion | ↑50% consumption |
| Membrane separation | Post-combustion | ↑31% consumption |
| PSA (solid sorbent) | Post-combustion | ↑32% consumption |
| TSA (solid sorbent) | Post-combustion | ↑42% consumption |
| Pre-combustion CCS | Fossil combustion | Blue water only |
| DACCS | No combustion | 4.01 m³/t CO₂ (range: 2.00–6.83) |
